# Editorial: Recent Advances on the Multimodal Search for Markers of Treatment Response in Affective Disorders: From Bench to Bedside?

**DOI:** 10.3389/fpsyt.2019.00790

**Published:** 2019-10-30

**Authors:** Frank M. Schmidt, Christian Sander, Martin Walter

**Affiliations:** ^1^Department of Psychiatry and Psychotherapy, University of Leipzig Medical Center, Leipzig, Germany; ^2^Clinical Affective Neuroimaging Laboratory, Otto von Guericke University of Magdeburg, Magdeburg, Germany; ^3^Leibniz Institute for Neurobiology, Magdeburg, Germany; ^4^Department of Neurology, Otto von Guericke University of Magdeburg, Magdeburg, Germany; ^5^Center for Behavioral Brain Sciences, Otto-von-Guericke-Universität Magdeburg, Magdeburg, Germany; ^6^Max Planck Institute for Biological Cybernetics Tübingen, Tübingen, Germany; ^7^Department of Psychiatry, University Hospital Tübingen, Tübingen, Germany; ^8^Department of Psychiatry and Psychotherapy, University Hospital Jena, Jena, Germany

**Keywords:** Depression, bipolar disorder, Editorial, Neuroinflammation, therapy prediction

Differentiated pharmacotherapeutic and psychotherapeutic antidepressive options are available, and an increasing number of patients receive treatment pursuant to evidence-based guidelines. Nonetheless, a large proportion of depressed patients show no or only partial response to initial interventions. This could improve if treatment were individually tailored toward the most promising options. Numerous advances in research have revealed that molecular, structural, and physiological alterations can bring about affective disorders. The use of such biomarkers and individual response predictors for antidepressant and antimanic interventions would be a major step toward truly personalized medicine and away from the prevailing trial-and-error approaches. This Research Topic consolidates and highlights state-of-the-art research on target pathomechanisms and the discovery of markers that forecast the outcome of therapeutic interventions, symptom severities, and the courses of affective disorders.

In their review, Herzog et al. delineate the discrepancy between the great efforts that have been made in the search for clinically useful biological predictors of antidepressant response on the one hand, and the limited advances that have resulted on the other. The authors put forward several compelling suggestions that could help to overcome major methodological drawbacks in therapy prediction research. As such, they suggest the use of animal models, interdisciplinary collaboration, and the application of multidimensional diagnostic criteria. Work by Lieb et al. suggests that brain-derived neurotrophic factor (BDNF) could be a candidate for therapeutic response prediction in clinical routine. The authors show that remission rates for antidepressant treatments are associated with methylation of the CpG-87 site of the BDNF gene. Further, in forecasting response prediction, methylation of the CpG-87 site and an early increase in plasma BDNF levels could add considerably to the clinically established predictors for early improvement. The review by Menke focuses on the undiminished potential of the hypothalamic–pituitary–adrenal axis. He considers alterations of the hypothalamic–pituitary–adrenal axis function both as a pathogenic origin of depression and as a promising target site for future treatment options beyond the monoaminergic systems. Hartmann et al. focus on the role of the autonomous nervous system (ANS) in major depression by investigating heart rate variability. Before treatment, depressed subjects showed a dysregulation of parameters indexing the parasympathetic branch of the ANS. Normalization of those parameters during the early course of therapy was associated with clinical improvement. These findings suggest that monitoring ANS activity (represented here by heart rate variability) as a state-dependent trait could improve diagnostic sensitivity and thus the therapeutic process. Depping et al. present the anatomical and functional properties of the cerebellum not only as an origin of psychomotor disturbances, but also as a participator in cognitive and self-referential processes that are relevant in affective disorders. To gather more information in this promising field, the authors propose important steps for future cerebellum-optimized analyses. The review by Himmerich et al. provides a comprehensive overview of how and where cytokines — important mediators of inflammation — are involved in depressive disorders. One noteworthy feature of the report is its emphasis on the pitfalls and yet unresolved questions that accompany cytokine research. Consequently, research results on this group of molecules remain unspecific and are vulnerable to a range of confounders. Beyond cytokine activity, the review by Culmsee et al. broadens the scope of inflammatory processes in affective disorders by outlining perturbations of mitochondrial and microglial pathways. Furthermore, the review issues compelling suggestions for novel yet unpursued targets within the immune and inflammatory system in both the treatment and early prognosis of psychiatric disorders. The investigation by Schmidt et al. further substantiates the link between inflammatory processes and the manifestation of depressive disorders. The authors describe a positive relationship between serum levels of cytokines, severity of depression, and extent of neuroticism. Moreover, proinflammatory cytokines appear to mediate the prediction of depression severity by the degree of neuroticism. These findings might encourage medical practitioners to discern whether a patient might benefit more from anti-inflammatory treatment strategies rather than conventional antidepressant therapies. The report by Reininghaus et al. reveals differences between sexes in the dynamics of tryptophan and kynurenine levels during the course of a psychiatric rehabilitation program. The findings suggest that sex may be a determinant for the relationship between tryptophan breakdown and clinical response. The report provides a basis for further research into how the tryptophan/kynurenine metabolism interacts with variations in sex-specific clinical features and how they could be considered in tailored treatment regimes. Bartkiene et al. propose a computer algorithm that registers and assesses facial expression responses to foods, a potential contactless option for predicting impending depressive episodes or for detecting them early on. Zheng et al. present an algorithm that utilizes both clinical and neurotrophic features. Their algorithm could improve the detection of bipolarity at an early stage of the disease, in turn improving the differential diagnosis of depressive episodes within a major depression or bipolar affective disorder. Schiller outlines different methods of quantitative electroencephalography (EEG) that could help in predicting antidepressant treatment response and support the choice of treatment. Beyond describing the methodological drawbacks that currently prevent these different algorithms from entering into clinical routine, he presents machine learning approaches that could potentially overcome these inconsistencies and thus come to be of interest in future clinical practice. Using a machine learning approach, Jaworska et al. identify features, prior to and at an early stage of therapy, that are early predictive of a response to a 12-week antidepressant trial in subjects with major depression. These findings show promise that combining demographic variables, scalp-level EEG power, and source-localized current density could improve predictive power in this cluster of entities and heterogeneous clinical presentation. Dorow et al. consider that the success of an antidepressant treatment strategy may strongly depend on the patients’ confidence in and attitude toward the therapy. In their investigation, perceived self-efficacy and educational attainment strongly affected patients’ choice of treatment. Intriguingly, secondary to psychotherapy, internet-based strategies were chosen equally as often as conventional medication.

In conclusion, the articles presented in this Frontiers Research Topic — diverse in their approaches and methods — provide a captivating overview of state-of-the-art research on pathomechanisms of affective disorders and predictors for successful treatment and give rise to the hope that some of these techniques will gain entry into clinical practice in the future.

## Author Contributions

FS wrote the initial draft of the Editorial. CS and MW critically revised the manuscript. All authors approved the submitted version.

## Conflict of Interest

The authors declare that the research was conducted in the absence of any commercial or financial relationships that could be construed as a potential conflict of interest.

